# Exosomal secretion of truncated cytosolic lysyl-tRNA synthetase induces inflammation during cell starvation

**DOI:** 10.15698/cst2018.05.137

**Published:** 2018-05-08

**Authors:** Sang Bum Kim, Seongmin Cho, Sunghoon Kim

**Affiliations:** 1 Medicinal Bioconvergence Research Center, Seoul National University, Suwon, South Korea.; 2 Department of Molecular Medicine and Biopharmaceutical Sciences, Graduate School of Convergence Science and Technology, Seoul National University, Seoul, South Korea.

**Keywords:** lysyl-tRNA synthetase, exosome, syntenin, caspase-8, inflammation

## Abstract

Previous work by Kim, *et al*. (2017) unveiled that lysyl-tRNA synthetase (KRS) is secreted through a mechanism involving syntenin-containing exosomes. They described how KRS, commonly known as part of the translational machinery in the cytoplasm, is secreted into the extracellular space where it induces inflammation. First, KRS secretion is triggered by starvation conditions. The increase in caspase-8 levels during starvation is responsible for proteolysis and generation of the N-terminal truncated form of KRS, and this event is required for KRS dissociation from the multi-synthetase complex (MSC). N-terminal cleavage of KRS eventually leads to a conformational change that allows its interaction with the C-terminal PDZ binding motif of syntenin and subsequent exosome biogenesis. The KRS-syntenin complex translocates to multivesicular bodies (MVBs) that originate from endosomes involved with intraluminal vesicle (ILVs). MVBs are transporters for the secretion of cellular contents into the extracellular space. Syntenin localizes intraluminal vesicles within endosomal membranes. The KRS-syntenin complex transfers on to intraluminal vesicles in MVBs. MVBs are translocated to the plasma membrane for ILV secretion mediated by Rab family proteins. Once KRS exosomes are secreted, their membranes are eventually ruptured by proteases and KRS is released from the exosomes where it can act as an inflammatory cytokine in the extracellular space. Secreted KRS triggers macrophage/neutrophil migration and induces inflammation.

Kim *et al*. (2017) reveals three crucial steps of KRS secretion, including its dissociation from the MSC complex, interaction with syntenin, and secretion via the exosomal pathway (**Figure 1**).

**Figure 1:**
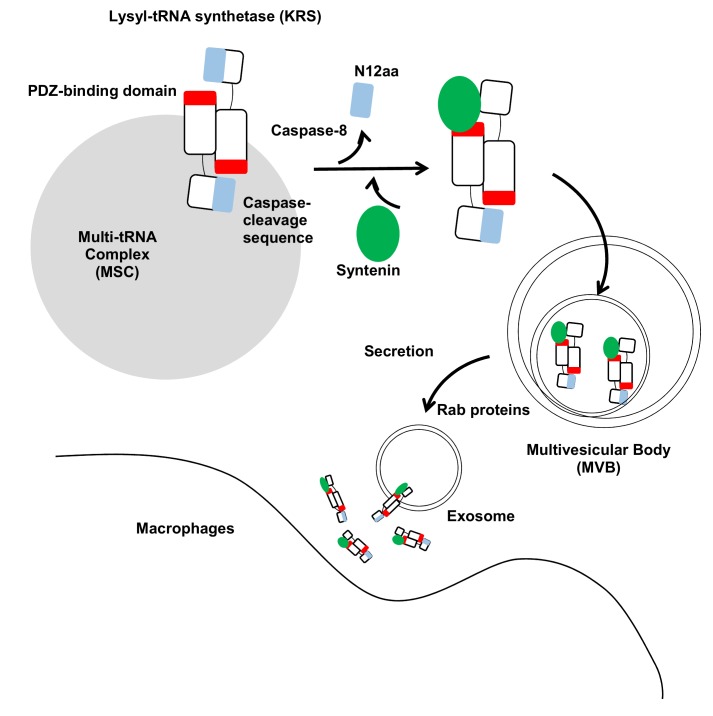
FIGURE 1: Schematic working model for KRS secretion. The two KRS monomers bind in an antiparallel fashion to form a homodimer. In this dimer, the N-terminal 12 aa of KRS (blue) may block the exposure of the PDZ-binding motif located at the C-terminal end (red) in the counterpart. The starvation-induced activation of caspase-8 results in cleavage of the N-terminal 12 aa to expose the PDZ-binding motif for docking of syntenin. Syntenin (green) binding causes the dissociation of KRS from the MSC and then translocates to the exosomes in multivesicular bodies (MVBs). MVBs are translocated to the plasma membrane for exosome secretion by Rab proteins. The KRS-containing exosomes are ruptured near macrophages, releasing KRS and triggering macrophage activation and migration.

First, syntenin is required for KRS exosomal secretion. This paper reveals how cytosolic proteins can transfer into exosomes via syntenin. Exosomal contents are composed of diverse cellular materials such as DNA, RNA, lipids, and proteins. Despite extensive evidence for the presence of cytosolic proteins within exosomes, the exact mechanism of secretion of cytosolic proteins remains unclear. The possible mechanisms include the use of chaperone proteins such as hsp90, hsc70, pkm2, and 14-3-3 epsilon that interact with exosomal membrane proteins and exosomal membrane lipid modifications including palmitoylation and myristoylation. Our data show that exosomal secretion of cytosolic KRS protein is induced through the syntenin-dependent exosomal secretion pathway. Kim *et al*. (2017) revealed that syntenin, an essential protein for exosome biogenesis, is required for cytosolic proteins to be secreted via exosomes. Although the complete mechanism is still unclear and further work is required, this study reveals a novel mechanism by which syntenin recruits cytosolic proteins for exosomes using its PDZ domain. The PDZ-binding motif is well known to interact with the PDZ motif. Therefore, we hypothesize that the PDZ motif and PDZ-binding motif interaction serves as one of the sorting mechanisms for exosomal secretion of cytosolic proteins. This mechanism would allow any proteins that have PDZ-binding motifs to be secreted into the extracellular space through exosomes.

The second key factor is caspase-8, which is known for its pro-apoptotic functions. The N-terminal region of KRS contains a caspase cleavage sequence, which is conserved in eukaryotic organisms. For KRS secretion, two conserved sequences in KRS, the N-terminal appended caspase cleavage sequence and the C-terminal PDZ binding sequence, are required. Caspase-8 cleaves the KRS N-terminal truncated sequence. This N-terminal truncated KRS induces the interaction with syntenin using the KRS C-terminal PDZ binding motif. Caspase-8 mediated KRS truncation causes tighter binding with syntenin. This indicates that KRS exosomal secretion is required for the interaction of these two conserved domains.

The final key factor involves starvation conditions. Many aminoacyl-tRNA synthetases (ARSs) have non-translational functions in diverse environments. Especially, KRS secretion is induced by starvation conditions. In starvation conditions, cellular protein translation is inhibited. As ARS is a component of the translational machinery, ARSs can perform non-translational functions such as inflammation and angiogenesis once translation is inhibited. When cells are challenged with mild cellular stress, the cells respond by activating reparative systems. By contrast, under severe cellular stress, the cells fail to repair themselves, leading to cell death. We suggest that cancer cells secrete KRS that promotes an inflammatory response when the cells experience translation inhibition, as occurs during serum starvation. We believe that the MSC or any ARSs, including KRS, could respond to cellular dysfunction and work to maintain cellular homeostasis. In the context of cellular dysfunction, we suggest that the function of KRS in the extracellular space is to promote inflammation and an immune response to either repair or kill the dysfunctional cells and maintain homeostasis.

KRS was shown to be secreted from cancer cells during starvation. When KRS secretion was induced, cellular caspase-8 levels were increased, but the pro-apoptotic protein caspase-3 was not altered. During apoptotic cell death, caspase-8 levels are increased followed by the activation of caspase-3 and caspase-8. During starvation conditions that promote the secretion of KRS, caspase-3 activation was not induced. However, when we incubated cells under starvation conditions for a longer duration, apoptosis was induced. After cell death, the immune system was activated to clear apoptotic cells. We hypothesize that KRS and other ARSs are cellular sweepers for the clearance of apoptotic cells. Before cell apoptosis, cells secrete KRS proteins into the extracellular space to facilitate clearance of dead cells following apoptosis. For apoptotic cell clearance, immune cell phagocytosis was increased, but KRS alone was unable to increase phagocytic activity in macrophages (data not shown). Therefore, we anticipate that some ARSs are secreted in starvation conditions, especially serum or amino acid starvation, and act to coordinate an immune system response to maintain homeostasis.

This work demonstrates that when cancer cells are confronted with cellular stress such as starvation in a microenvironment, the immune system can be activated by KRS, one of the ARSs. This suggests a functional link between the caspase signaling pathways, the exosome biogenesis system, and a key enzyme that functions both as part of the translational machinery and as a modulator of the immune system in the extracellular space.

